# How Parents Cope With Value Tensions in Their Young Child's Nutrition: An Interview Study Informed by Paradox Theory

**DOI:** 10.1111/mcn.70214

**Published:** 2026-07-01

**Authors:** Noa van den Brink, Marina Bos‐de Vos, Valentijn T. Visch

**Affiliations:** ^1^ Faculty of Industrial Design Engineering Delft University of Technology Delft the Netherlands

**Keywords:** children, coping, distress, facilitators, nutrition decisions, value tension, well‐being

## Abstract

When facing challenging nutrition decisions, parents experience tension between their personal values and those they have for their child. These tensions a risk of leading to unhealthy nutrition decisions, particularly in disadvantaged environments where resources for healthy nutrition are scarcer. Prioritising one value over another, i.e., a ‘trade‐off’ such as a child's dietary health over the parent's convenience, can cause stress due to unsatisfactory outcomes and recurring value tension. Drawing on Paradox theory, this qualitative study examines how parents cope with nutrition‐related value tensions. Paradox theory explains how individuals manage persistent tensions between competing values by adopting ‘both/and’ strategies that address both values simultaneously rather than choosing one over the other, i.e., ‘either/or’ strategies. In the present study, semi‐structured interviews were held with 20 parents of children aged 0–5 living in disadvantaged neighbourhoods. Using abductive thematic analysis, we identified coping responses classified as ‘either/or’ (value trade‐offs) and ‘both/and’ (compromise and paradoxical resolution). While trade‐offs were more common, paradoxical resolution strategies, those that address competing values simultaneously, were less frequently employed during challenging moments related to nutrition decisions. Three main themes of ‘both/and’ coping responses emerged from the analysis: planning and preparing food, managing stressful child behaviours, and supporting children's healthy food acceptance. Factors facilitating these coping responses ranged from individual, interpersonal, to community levels, including time availability, social support, and healthy routines encouraged by daycare. Our findings suggest that ‘both/and’ coping, aligned with parents' values and context, can support parents in managing food provision and feeding challenges while upholding their values, provided there are sufficient facilitating factors available.

## Summary

- Parents often cope with nutrition challenges by prioritising one value, such as nutritional health, which can cause distress.
- ‘Both/and’ coping responses, such as meal planning, managing child behaviour through play, and promoting healthy food acceptance via child autonomy, help address both parental non‐health values and child nutrition, reducing distress and increasing well‐being.
- Time, material resources, and social support facilitate ‘both/and’ coping; it is crucial that interventions and policy move beyond individual responsibility to address contextual barriers.
- Professionals should consider offering practical coping options tailored to parents' values and circumstances, while research explores which coping responses remain effective for promoting healthy eating over time.

## 1 Introduction

Despite parents' best intentions, providing healthy nutrition to their families and preserving their own well‐being can be difficult. For instance, a lack of child discipline often contributes to experienced parental stress and the use of unhealthy foods and feeding practices (Berge et al. 2017; Tate et al. 2020). In these moments, parents can experience tensions between the values they hold. A value is a guiding principle or belief that expresses what is considered good or desirable. Parents shape and evaluate their nutrition decisions based on their values (Damen et al. 2020; Schwartz 1992). Value tension arises when one value drives actions that undermine another (Smith and Lewis 2011). For example, soothing a child with snacks for their enjoyment may support parental well‐being but conflict with the value of promoting the child's nutritional health. Other values, such as affordability, convenience, enjoyment, and social relationships, can similarly challenge healthy nutrition choices (Hayter et al. 2015; Killion et al. 2023; Pescud and Pettigrew 2014; Schuler et al. 2024).

Contextual constraints, such as limited time, can lead parents to make nutrition decisions that prioritise one value, such as the convenience of food, over others, such as their children's nutritional health. While this may offer short‐term relief, unresolved value tensions recur and can lead to unfavourable outcomes (Folkman et al. 1986; Lewis 2000; Smith and Lewis 2011). Moreover, these unmet values can cause emotional distress in parents (Damen et al. 2020; Yue et al. 2025). For instance, prioritising a child's nutritional health over parental enjoyment of foods may support child health. Still, it can create ongoing stress for parents who do not address enjoyment, thereby affecting their personal well‐being. Parents often face value tensions between their child's dietary health and non‐health values, and have to cope with them. In this context, well‐being refers to the positive affect, such as feeling pleased or relieved, derived from the extent to which parents' values beyond dietary health, such as convenience, affordability, enjoyment for both parent and child, social belonging, and self‐care (van den Brink et al. 2025), are also fulfilled.

Paradox theory provides a framework for understanding how parents can cope with value tensions. It supports conceptualising a value tension as a paradox between two coexisting values that may be equally important yet difficult to reconcile (Lewis 2000; Smith and Lewis 2011). Coping responses can take the form of a value trade‐off, referred to as an ‘either/or’ coping in paradox literature. This strategy prioritises one value over another, such as choosing the convenience of quick meals, which may conflict with the ideals of a healthy meal. In contrast, ‘both/and’ coping aims to satisfy multiple values simultaneously. By doing so, parents can reduce distress associated with unmet values in their nutrition decisions. For example, preparing extra meals in advance offers healthy options for the child while saving time and offering convenience for the parent (Walsh et al. 2015). This ‘both/and’ approach reduces immediate cooking stress while also satisfying both convenience and the child's dietary health, preventing later distress.

Coping responses related to healthy nutrition, such as feeding practices, meal planning, and food preparation, are well‐studied. Alm and Olsen (2017) found that parents avoid preference conflicts by choosing accepted foods, planning convenience meals for busy days, or batch‐cooking. Walsh et al. (2015) reported responses, such as hiding vegetables in mashed foods or preparing healthy options for social occasions. However, current research does not explicitly identify which coping strategies address parents' underlying values regarding nutrition challenges, thereby enhancing favourable outcomes of nutrition decisions for parents. Using a paradox lens in nutritional decision‐making can enrich the literature by revealing the coping responses that support both the child's healthy eating and the parent's non‐health values.

The predominant approaches to parents' behaviour change in nutrition have focused on the concept of personal responsibility (Arlinghaus and Laska 2021; Brownell et al. 2017). However, focusing solely on parents' individual coping overlooks systemic and contextual barriers that shape nutritional choices (Jovanovski and Cook 2019). Supporting ‘both/and’ coping strategies, for example, through professionals or interventions, thus requires an understanding of how parents can implement coping responses within their specific circumstances. Contextual constraints in parents' circumstances are especially pronounced in disadvantaged areas, where limited time, support, and access to healthy foods constrict healthy options (Byrnes and Miller 2012; Vilar‐Compte et al. 2021). For example, Devine et al. (2006) found that low‐income working parents often had to rely on quick, less healthy meals to manage stress. The authors suggest that this contrasts with research on higher‐income couples, who can use strategies such as planning meals or taking time off for family care, due to differences in job conditions, including stable work schedules. Therefore, enabling ‘both/and’ coping through interventions requires considering not only individual choices but also interactions with parents' contexts. This approach provides insights for interventions to adapt to and address contextual constraints.

Accordingly, this qualitative study investigates the research question: how do parents of children aged zero to 5 years old in disadvantaged neighbourhoods cope with nutrition‐related value tensions, and how does their context influence these coping responses?

## 2 Methods

### 2.1 Study Design

We employed semi‐structured interviews to investigate how parents navigate nutrition‐related value tensions. This method is particularly suited to capturing lived experiences and allows for probing (Magaldi and Berler 2020), such as how contextual and socio‐ecological factors shape those experiences.

Building on the work of van den Brink et al. (2025) on value tensions parents experience in nutrition choices for their children, this paper examines the coping responses parents used to address these value tensions.

The present study is conducted as part of the Our Smart Family Buddy project. This 6‐year project, involving multiple scientific domains and stakeholders, aims to develop a platform to improve healthy nutrition choices and decrease the stress of young families in disadvantaged neighbourhoods. The project research protocol is available as a preprint authored by Wieles et al. (2025).

### 2.2 Participant Recruitment

Parents were eligible to participate if they resided in a designated disadvantaged area of Rotterdam in the Netherlands, were at least 20 years old, and were the primary caregiver for a child aged 0–5 years old. Recruitment took place between June and October 2023 through key community members, local events, public spaces such as shopping centres and playgrounds, and WhatsApp groups. Both biological parents and primary caregivers (e.g., grandparents) were included and are hereafter referred to as ‘parents.’ Convenience sampling was used, with participants invited after expressing interest. In households where both partners participated, these parents were counted as one participant. Each participant received a €20 gift card.

The selected neighbourhood for recruitment has a socioeconomic status score of −0299, below the national average of zero (Centraal Bureau voor de Statistiek 2024). Among residents, 55% have a migration background, 15% is a single parent household, 37% is classified as lower educated (ISCED 1–2), and 13% live at the social minimum (AlleCijfers, & CBS 2025; Centraal Bureau voor de Statistiek 2023; Gemeente Rotterdam 2022).

### 2.3 Data Collection

The participants chose the interview locations based on their preferences. Locations included participants' homes, cafés, community centres, and parks. The participant's children were often present. Interviews were conducted by one or two researchers. When two researchers were present, the second researcher monitored the depth of the conversation by ensuring that sufficient follow‐up questions were asked and kept track of time. Interviewers had no prior relationships with the participants. Interviews lasted between 60 and 90 min.

To support reflection, participants completed preparatory exercises about their nutritional habits and emotionally challenging situations before the interview (Additional file 1). These exercises, sent on WhatsApp, helped participants enter the interview with deeper insight into their experiences (Visser et al. 2005). The interviews explored challenging moments related to child nutrition and the coping responses participants employed to address them. These moments reveal underlying value tensions, as evidenced by the parents' expressed distress, including struggles, concerns, or feelings of regret (Damen et al. 2020).

The interviews began with a discussion of the preparatory exercises, followed by image cards depicting nutrition, family life, and values, which were developed from informal parent dialogues and literature. For example, prior research on snack‐related conflicts before dinner (Damen et al. 2020) informed the selection of relevant images. If the preparatory exercises were incomplete, interviews began with open‐ended questions, then moved to browsing the cards and identifying any that resonated with parents' experiences, allowing them to elaborate on them. Interview questions included: “What are difficult moments regarding food in your family?”; “How do you handle such moments?”; “Can you provide examples?”; “Who else plays a role in this moment?”; “What are you proud of in how you handled difficult situations related to nutrition?”. See additional file 2 for the complete interview guide. In addition to these open questions, participants were also asked to provide demographic information during the interview. Data were collected through audio recordings and transcribed verbatim. Most interviews were conducted in Dutch, except for two in Arabic and Turkish, where an interpreter and a live translation app were used, respectively.

### 2.4 Data Analysis

We employed an abductive approach to thematic analysis (Thompson 2022), combining inductive and deductive methods to examine how parents coped with the value tension between the child's dietary health and the parent's non‐health values (Convenience, affordability, enjoyment for both parent and child, social belonging, and self‐care) in challenging nutrition‐related moments. To investigate coping strategies and responses, this study used the dataset and analysis from van den Brink et al. (2025) on the value tensions young families experienced.

The first author initially open‐coded four transcripts, focusing on parental coping behaviours and their contextual influences in relation to pre‐identified value tensions. After discussing these codes with the research team, they collaboratively developed a preliminary codebook, which was iteratively refined as the first author coded the remaining transcripts. Next, emergent coping response codes were mapped during the deductive coding phase onto categories of ‘either/or’ or ‘both/and’ coping strategies, informed by Paradox theory (Smith and Lewis 2011, 2012). This involved identifying whether a coping response prioritised a single value as a trade‐off ('either/or coping’) or reconciled both the nutritional health of the child and non‐health values (‘both/and coping’). The latter was subdivided into ‘compromise’ and ‘paradoxical resolution’ strategies due to their differences in the degree to which they address both values, as outlined in Table 1. ‘Either/or’ coping was identified if the participant described the situation as still causing distress or the coping response was not preferred. ‘Both/and’ coping was identified if parents referred to coping responses with terms like ‘avoiding stress’, ‘this is fine, instead of…’, or ‘better’, or if they described a coping response in relation to questions about what is currently working well for them, indicating it addressed or avoided distress and supported parent well‐being. The coping response was coded as a compromise strategy if it integrated options of satisfying the health of the child and a non‐health value, but left one or both values not fully satisfied. Paradoxical resolution was coded if the coping response resolved or prevented distress by having a solution that fully addressed both values. All final code groups were organised into themes reflecting the purposes of parents' coping responses, such as planning ahead or managing challenging child behaviours. In these final themes, we focus solely on ‘both/and’ coping responses, rather than ‘either/or’ responses, to identify strategies that address both values involved in value tensions during nutrition decisions and the conditions under which they apply, rather than prioritising one value over another. A classical content analysis was conducted to quantify the frequency and extensiveness of the found either/or (trade‐off), and both/and (compromise and paradoxical resolution) categories of coping responses (Bauer 2000), capturing how often each response was used and the number of participants who used it. The unit of analysis was each distinct coping response. The sub‐theme ‘coping facilitators’ was mapped onto the socio‐ecological model, encompassing individual, interpersonal, and community levels, drawing on the work of Rios et al. (Bronfenbrenner 1977; Rios et al. 2024). Data saturation was reached when no new themes emerged in the final set of interviews. Analysis was conducted using Atlas.ti software (ATLAS.ti Scientific Software Development GmbH 2024).

**Table 1:** Descriptions of coping strategies and their corresponding categories based on Smith and Lewis (2012).

Coping strategy category	Coping strategy	Description
Either/or coping	Value trade‐off	Making a nutrition decision that prioritises meeting one value of the parent over another.
Both/and coping	Compromise	Making a nutrition decision that meets both values to a limited extent, so that one or both values are only partially fulfilled.
	Paradoxical resolution	Making a nutrition decision that fully meets both competing values.

### 2.5 Ethics Statement

The study protocol was approved by the Human Research Ethics Committee TU Delft (approval number 3420). Written informed consent was obtained from participants before the interview. Participation required written informed consent, and all participants received financial compensation for their time. The research was conducted in accordance with the Netherlands Code of Conduct for Research Integrity.

## 3 Results

### 3.1 Participant Characteristics

The study involved 18 families, comprising a total of 20 individual parents. A total of 17 interviews were conducted, comprising 14 individual interviews and 3 paired interviews. Two paired interviews involved parents from the same household, one included parents from different families. A summary of participant characteristics is provided in Table 2.

**Table 2:** Participant characteristics.

Participant characteristics	*N* = 20 (%)
Age	
Median age	39 years
Age range	24–57 years
Not specified	2 participants (10%)
Gender	
Woman	18 (90%)
Man	2 (10%)
Highest level of education, ISCED 2011 (UNESCO Institute for Statistics 2012) (UNESCO Institute for Statistics 2012)	
Low education (ISCED 0–2)	5 (25%)
Medium education (ISCED 3–4)	2 (10%)
High education (ISCED 5–8)	10 (50%)
Not specified	3 (15%)
Job status	
Paid job	10 (50%)
No paid job	7 (35%)
Studying	1 (5%)
Not specified	1 (5%)
Marital status	
Single‐parent household	4 (20%)
Two‐parent household	16 (80%)
Youngest child's age	
Median age	1 year
Age range	0–5 years
Child age (entire family)	
Median age	4 years
Age range	0–15 years

### 3.2 Coping Strategy Occurrence

The interview analysis revealed diverse coping strategies parents used to manage value tensions during challenging moments that shaped their children's nutrition choices. For example, a challenging moment occurred when treats were brought home from daycare or by family members, creating a tension between supporting the child's enjoyment and safeguarding their nutritional health. This affected parents emotionally through distress, contributing to tension between their own non‐health values and the child's nutritional health.

Participants described coping responses fitting three main coping strategies: ‘value trade‐off,’ ‘compromise’, and ‘paradoxical resolution’ (see Table 3). ‘Value trade‐off’ is ‘either/or’ coping, while compromise and paradoxical resolution are ‘both/and’ coping. For example, when a child wanted a bag of candy, one parent chose a trade‐off by allowing the child to eat it, prioritising enjoyment over health. Another parent compromised by offering a small portion to balance both values. A paradoxical resolution involved providing an alternative snack that was perceived as both healthy and enjoyable. Another example concerns coping with children's food rejection. One parent made a trade‐off between convenience and health by promising sweets after dinner as a convenient way to get their child to eat, whereas another made a compromise on enjoyment and nutritional health by swapping foods the child wouldn't eat for alternatives that provided enjoyment, even though they had less nutritional value. Lastly, a parent used paradoxical resolution by encouraging a consistent sleep routine for their child to reduce food rejection behaviours, creating an opportunity to meet both the child's enjoyment and nutritional health.

**Table 3:** Coping strategy occurrence across participants and in total.

Coping strategy	Value trade‐off (‘Either/or’ coping)	Compromise (‘Both/and’ coping)	Paradoxical resolution (‘Both/and’ coping)
Number of participants employing it	18 (100%)	17 (94,4%)	14 (77,8%)
Total occurrence	77	66	34

For the value tension between the dietary health of the child and parental non‐health values, paradoxical resolution was the least frequently mentioned response, as parents more often reported making a choice between values (trade‐off) or partially accommodating both (compromise). Nevertheless, 77.8% of parents and couples referenced paradoxical resolution at some point, indicating widespread use despite lower mention frequency in challenging moments. This suggests that while most parents have employed a paradoxical resolution strategy, they often struggle to find or implement suitable resolutions in challenging moments.

### 3.3 ‘Both/and’ Coping Responses

**Figure 1:**
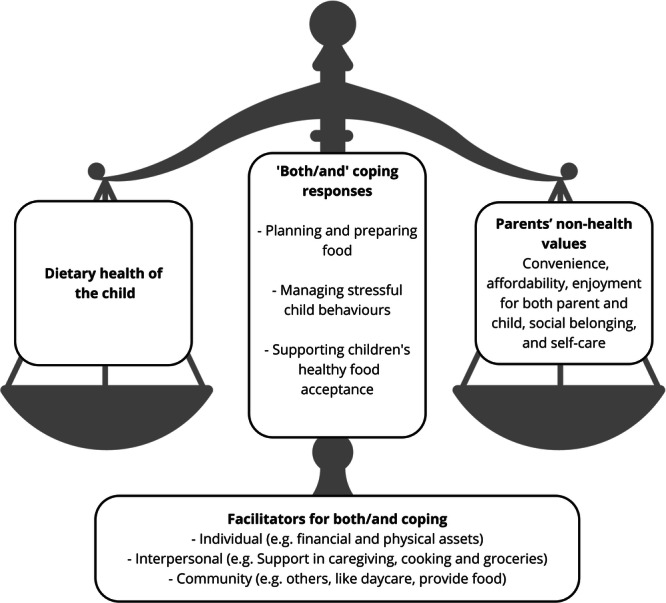
Visual representation of the ‘Both/and’ coping responses and their facilitators (adapted scale vector from source: www.vecteezy.com).

This section examines the coping responses that enabled parents to navigate challenging moments in nutritional decisions for their child by successfully addressing two values in tension, positively influencing the nutritional health of the child and the parents' non‐health values, contributing to well‐being (Figure 1). We found three overarching responses of ‘both/and’ coping: planning and preparing food, managing stressful child behaviours and supporting children's healthy food acceptance. Additionally, facilitators were identified that made these coping responses possible for parents. See an overview of all themes, related coping responses and their facilitators in Table 4.

**Table 4:** Overview of ‘Both/and’ coping themes, sub‐theme responses and coping resources.

‘Both/and’ coping themes	Coping responses	Coping facilitators
Planning and preparing food	Planning of groceries	Individual Available time Financial and physical assets Interpersonal Tips on quick and healthy foods Support in caregiving, cooking and groceries CommunityOthers, like daycare, provide food
	Preparing food in advance	
	Preparing healthy food swaps	
Managing stressful child behaviours	Managing food provision strategies Giving healthy food options Eating snacks without the child Compromising the amount of undesired food	Individual Storage of healthy food alternatives Interpersonal Child also enjoys healthy food alternatives given Information on dealing with difficult behaviour Community Routine through daycare and school Daycare and school policy on healthy eating Play opportunities for the child
	Entertaining and distracting the child without food Playing with the child Screen time	
	Retaining routine and rules for the child	
Supporting children's healthy food acceptance	Adapting food Changing food structure Swapping rejected food for accepted food	Individual Information on recipes and picky eating Feeling secure about the child's weight Interpersonal Positive signs of food acceptance of the child Community Different environments and people supporting the child's eating
	Supporting child autonomy in eating Supporting the child's choice in which foods are prepared Trial and error in food acceptance Compromising healthy food amount Compensating with supplement usage	
	Parent role modelling	
	Retaining routine for the child	

### 3.4 Planning and Preparing Food

Parents faced challenges in providing healthy, affordable, and convenient meals or snacks that their children would also enjoy at home, on the go, or at daycare. Coping responses around food planning helped avoid situations in which the child's nutritional health, along with other concerns driven by values such as affordability and convenience, would not be met.

#### 3.4.1 Coping Responses

Parents employed both compromise and paradoxical coping strategies in planning and preparing food. Parents used grocery planning to manage costs and ensure the availability of ingredients for preparing meals in a timely and healthy manner as a paradoxical resolution. Some structured meals around recipes or leveraged store discounts and market prices, while others opted for flexible ingredient choices. For three parents, their child's preferences significantly influenced food budgeting decisions. One mother, for instance, prioritised her youngest child's tastes to encourage food acceptance, balancing this with her perception of healthy eating as a compromise.

No, we mainly focus on what he [youngest child] needs. We pay particular attention to him. So with the rice cake, that's something he eats.

(Participant 15)

To reduce stress and ensure meal timeliness, three parents indicated that they prepare meals in advance by cooking in larger batches or prepping ingredients. These practices helped reduce the stress of last‐minute cooking, especially during busy or stressful times, and aligned with their values of self‐care and convenience. For example, prepping meals the day before mitigated the stress created by children's crying, enabling healthy meals without cooking stress as a paradoxical resolution:

That's why I always make sure the day before that there's something set aside for him for the next day. And I do that every day, preparing for the following day, to avoid stress. Because when he's hungry, as you just heard, he switches on [crying]. And he doesn't switch off until he's eaten something. I know I can handle that for about 10 min, but I can't deal with it if I still have half an hour of things to prepare.

(Participant 8)

Lastly, as a paradoxical resolution, parents planned ahead by preparing healthy snacks and drinks for situations at home, daycare, or on the go. This helped avoid giving children unhealthy options, either by others or due to a lack of alternatives, and reduced the need to buy expensive snacks while out.

Sometimes they [daycare] hand out ice creams in the summer. We always give our own ice cream [to daycare], just homemade from juice.

(Participant 13)

#### 3.4.2 Coping Facilitators

Parents' ability to buy groceries or prepare meals on time depended on their available time, which was influenced by their employment status, caregiving responsibilities, such as whether these were shared with a partner or others, and other obligations, including appointments to the hospital or municipal offices. Participant 2 experienced no time to go to the market due to these appointments, making it difficult to buy all the desired food items at the supermarket, where prices were higher than at the market.

P2: Sometimes I don't have time to go to the market. Then I just go quickly, quickly. That's really the big difference.

Interviewer: […] And is that still manageable financially?

P2: Difficult, really difficult. Then you have to skip something. […] If you go [to the market], everything you want, you will buy.

(Participant 2)

Financial and physical resources, such as income and freezer space, enabled bulk shopping and food ordering, easing time and monetary pressures. Furthermore, parents who received support with childcare, cooking, or groceries from partners and family members experienced less strain. For example, a parent kept the children entertained while their partner or a grandparent cooked without interruption, and another relied on their partner to prepare meals when feeling tired:

So yes, if I don't make the meal, then he [my partner] makes it. If I'm tired, then he makes it.

(Participant 7)

One parent found tips about quick and healthy meals from her mother and friends helpful in easing mealtime difficulties. Lastly, daycare or kindergarten services contributed to structured eating routines by providing healthy meals, reducing parents' stress in providing them.

And just like, eating healthy when you've had a busy day. And then one evening you have fries. But they've eaten healthy all day at daycare.

(Participant 14)

### 3.5 Managing Stressful Child Behaviours

Parents experienced stress when their child requested undesirable foods or displayed challenging behaviours, such as crying or ignoring requests or set rules. These moments made it difficult to make healthy nutrition decisions while also ensuring the child's enjoyment, managing behaviour conveniently, and protecting the parent's self‐care. Parents alleviated this stress by managing food provision, creating other distraction strategies, or maintaining a daily routine.

#### 3.5.1 Coping Responses

To manage nagging and tantrums, parents used food as a response. Children often requested snacks, especially before dinner, on holidays, and on weekends, making it harder to maintain a healthy diet. Firstly, this involved a compromise strategy. Parents frequently compromised on snack demands by serving smaller portions or diluting sugary drinks to pacify children or avoid feelings of guilt. To ensure children still ate dinner, snacks were given later, such as after meals.

Paradoxical resolutions were also evident in parental coping responses aimed at managing stressful child behaviours. Three parents avoided nagging by eating their own snacks out of sight, such as in the evening or outside the home. Additionally, some offered healthy snacks at home, though options were limited by storage and the desire for variety.

And at 9:30, he's already saying he's hungry again. Then he wants to eat something again. So I'll give him a cracker, for example. Yeah, and then 45 min later, he asks again, saying he's hungry. Then it's another piece of fruit.

(Participant 5)

Some parents practised paradoxical resolution by avoiding giving undesired foods while involving children in play. Playing outdoors and indoors was considered effective in managing children's energy and lowering parents' stress. One parent also observed that their child ate better after playing outside:

Playing. If he wants to play outside, then he eats better.

(Participant 4)

Screen time was another distraction used during dinner, when tantrums occurred, or while cooking. This paradoxical resolution helped alleviate stress and freed up the parent to focus on cooking. One parent noted the benefit that her child ate more when watching screens:

Watching TV or the phone. If he keeps watching, then he eats a lot.

(Participant 17)

Maintaining routines, such as setting consistent bedtimes, mealtimes, and clear food rules across family members, such as partners, was perceived as helpful coping. It helped children listen better, accept healthy foods, and reduce tantrums that could lead to giving in to unhealthy options. This way, it functioned as a paradoxical resolution.

#### 3.5.2 Coping Facilitators

Parents' ability to offer healthy snacks to reduce nagging and tantrums depended on having enough variety and storage of healthy snacks at home, which is linked to the coping response of food planning. Six parents actively sought advice on managing their children's challenging behaviours, such as tantrums or crying, through social media, including YouTube and Instagram, as well as conversations with other parents. Some parents tried out these tips, leading to positive outcomes for themselves, such as improved child listening and greater confidence in handling child behaviour and finding calm.

And I also look on YouTube for people who talk about nutrition. […] Because I had problems with my oldest daughter. When she says no and gets a bit difficult, she cries. I have to stay firm. And I also get angry. They really helped me with parenting. I just need to step outside for a moment and breathe.

(Participant 10)

Four parents noticed positive signs that their child enjoyed healthy foods, such as enthusiasm for eating a vegetable, which encouraged them to offer these foods during moments when their child asked for food or had a tantrum.

That one [child] no longer asked for sweets and things like that all the time. And then I think, well, just enjoy a piece of bell pepper or something, if you're just as enthusiastic about that.

(Participant 13)

Parents viewed daycare and school routines as supportive in ensuring healthy eating, as they created a routine for their child's eating and limited stressful moments for parents about what to offer their child. Additionally, policies such as banning unhealthy foods or sending treats home instead of serving them at daycare made it easier to maintain healthy habits and decide when treats were appropriate. However, some parents felt these rules limited their autonomy in choosing what was best for their child. In the community, parents noticed that access to safe, nearby play areas also supported healthy eating and reduced challenging behaviours. However, five participants lacked sufficient space at home or nearby safe play options.

There are a lot of [playgrounds], but they need maintenance. And some aren't very safe for small children. They can easily walk into the street. And for older children, there also isn't much to do, which leads them to start misbehaving.

(Participant 15)

### 3.6 Supporting Children's Acceptance of Healthy Foods

Parents faced challenges when their children rejected foods perceived as healthy. This made it difficult to support their child's nutritional health while also ensuring the food was enjoyable and convenient to offer.

#### 3.6.1 Coping Responses

To help children accept rejected foods or try solids, some parents relied on compromise responses. They swapped disliked foods for alternatives that were both accepted and enjoyed, as it was essential to these parents that their child would still eat something, such as by preparing a different version of the meal or offering a sandwich:

Sometimes, for example, if someone doesn't want to eat fruit, because sometimes he doesn't, then I give him a sandwich.

(Participant 10)

Others supported children eating by encouraging them to take a few bites of disliked foods or allowing them to choose between options for dinner. Parents who felt uncertain about their child's nutrition sometimes compensated with supplements or bottle feeding, balancing enjoyment with nutritional needs:

The oldest one is difficult with food. They only eat bread, or sometimes not even that. I just give vitamins.

(Participant 3)

Paradoxical resolutions were also used to address both values simultaneously. Four parents adjusted the texture of their children's food, such as blending or cutting vegetables, to encourage acceptance of healthy foods, supporting the value of the child's enjoyment. Some offered choices within healthy options, assisting children to engage more willingly. Persistence was another approach, with parents reintroducing rejected foods later until signs of acceptance appeared. A few parents emphasised role modelling, adapting their own diets to include both healthy and enjoyable foods, to encourage healthy eating and justify their expectations for their child. Another parent noticed that her child eats better when they sleep well. Finally, having a daily routine to ensure timely sleep was experienced as a valuable coping response to help their child accept healthy foods.

And I also find structure really important. That's mainly because when you have children, you simply need it. You need a set time for going to bed and for having eaten on that time, so they can still play a bit and then go to bed tired. […] And he becomes very annoying if he's not in bed by seven. And especially because it then affects his difficulties with eating. He usually eats everything, but he's a picky eater when he's tired, so to speak.

(Participant 14)

#### 3.6.2 Coping Facilitators

Parents found recipe ideas and coping tips, such as introducing solids or managing picky eating, helpful for trying new healthy meals and coping responses. They selectively used information based on their child's preferences, convenience, affordability, and the child's level of autonomy in eating that would make the parent feel secure. Sources included family, daycare providers, health professionals, parent groups, the internet, and social media platforms such as Facebook.

There are websites in this space; mom‐focused sites, baby food sites, and so on. From there, I look at things and sometimes try out the recipes. If he likes it, I go for it. I try different things like that.

(Participant 1)

Additionally, one parent explained that maintaining their child's autonomy in eating depended on feeling secure enough to experiment with this, particularly in relation to the child's weight.

So, actually, you dare to experiment with it. Yes, I mean, you always can… they're not skin and bones. Not at all. So you can always just go back. Yes, that's another thing.

(Participant 13)

Environments like schools and daycares encouraged children to try new foods and helped parents see that their child could enjoy specific healthy options. This boosted their confidence to offer the same foods at home. For example, one parent felt discouraged when her child rejected bread, but gained confidence after a successful feeding experience at daycare.

Because a few months ago, he wouldn't even eat bread. And I was really worried about that, because for a long time, he would gag whenever he ate bread. […] And then at some point, I heard back from the daycare… He ate the whole sandwich. Okay, great. That's nice. So now I'm doing the same with fruit and everything else. I'm just going to keep offering it.

(Participant 8)

## 4 Discussion

### 4.1 ‘Both/and’ Coping Strategies for Dietary Health of the Child and Parents' Values of Convenience, Affordability, Enjoyment, Social Belonging, and Self‐care

This study's findings revealed that parents in disadvantaged neighbourhoods use various ‘both/and’ strategies in coping responses to navigate nutritional challenges. These responses are eligible to help them address the value of the nutritional health of their child and avoid the distress of outcomes that are inconsistent with another value they hold as a parent (Folkman et al. 1986). While previous research has identified coping related to planning and preparing food, managing stressful child behaviours, and supporting children's healthy food acceptance (Ahluwalia et al. 1998; Hayter et al. 2015; Jawad et al. 2025; Schuler et al. 2024; Walsh et al. 2015), we contribute by demonstrating how specific, situated responses can be supportive to both parents' non‐health values (Convenience, affordability, enjoyment for both parent and child, social belonging, and self‐care) and the child's nutritional health in self‐identified challenging moments. Some particular coping responses parents reported include preparing foods in advance, distracting the child without food, and supporting the child's autonomy in trying foods. For instance, preparing healthy foods that the child is known to accept supported both the child's dietary health and enjoyment, while addressing the stress associated with food rejection and conflict that can affect parental well‐being. ‘Both/and’ coping as addressing distress was mainly analysed from a theoretical perspective and through some direct parent statements indicating relief. Future research may further investigate the effects on distress.

Although our findings align with patterns observed in broader food provision research, the ‘both/and’ strategies should be viewed as exploratory and shaped by a specific socioeconomic context. Therefore, any connection to broader parental populations should be considered tentative and may vary based on factors such as economic and interpersonal support resources, neighbourhood influences, such as access to healthy nutrition, and personal and cultural norms related to nutrition choices.

As part of ‘both/and’ coping strategies, parents employed compromises and paradoxical resolutions, each addressing competing values to varying degrees. Paradoxical resolutions, where both values are engaged simultaneously, were mentioned less frequently than compromise and trade‐off strategies. Paradoxical resolution strategies, however, appear promising, as they often alter challenging situations to alleviate tension. For example, some parents managed behaviour through compromise by offering smaller portions of unhealthy foods. In contrast, paradoxical resolution approaches, such as incorporating playtime, supported both the child's dietary health and convenience for the parent or enjoyment of the child: play reduced disruptive behaviours and improved food intake. Prior research has linked physical activity to healthy diets in children (Gubbels et al. 2013), though evidence on play, considered a form of physical activity, as a form of emotion regulation remains mixed (D'Cruz et al. 2024). Nevertheless, such paradoxical resolution strategies reflect problem‐focused coping, which can prevent stress by altering the situation, consistent with Folkman's coping theory (Folkman 1984; Folkman et al. 1986). Other examples in our results of coping responses as paradoxical resolution strategies include batch cooking, maintaining routines, and encouraging child autonomy in healthy food choices.

Some responses using ‘both/and’ coping strategies, however, diverged from healthy lifestyle recommendations, such as the use of supplements to compensate for unhealthy foods. Some coping responses also affect other lifestyle domains beyond nutrition, such as screen use to occupy children during cooking or dinner, which may create new tensions over time, given its link to developing socioemotional problems (Vasconcellos et al. 2025). Therefore, these coping responses are maladaptive and may not provide a paradoxical resolution over time. Our research identified ‘both/and’ coping responses from the parents'’ momentary perspective. This can help explain why a certain maladaptive coping response is adopted, as it benefits a parent's values in the moment and within their current means. For future research, we recommend examining ‘both/and’ coping responses, that address both the child's dietary health and parental non‐health values, while aligning with healthy lifestyle recommendations. Thus, creating lifestyle advice that does not add to felt tension, but supports integration with parental values. For example, advice that helps integrate the values of convenience, the child's enjoyment, or the parent's self‐care. Additionally, it would be beneficial to study ‘both/and’ coping over time and across lifestyle domains, not just in nutrition.

### 4.2 How to Tailor Interventions to Support Parents in ‘Both/and’ Coping Strategies

Parents reported adopting coping responses after experiencing success. For example, when a child accepted a previously rejected food in a different setting, parents felt more confident to offer it at home. Prior research also shows that children observing peers eating can encourage them to try new foods (Hayter et al. 2015; Lovelace and Rabiee‐Khan 2015). We therefore suggest studying how coping responses can produce observable positive outcomes, such as signs of food acceptance or improved child obedience, to motivate parents to try or continue using them.

Our findings also indicate that some parents chose specific information on managing food rejection based on their child's preferences, convenience, affordability, and the desired level of autonomy of their child in eating. Similarly, other studies highlight the need for practical, tailored advice that accounts for differences among children, family contexts, and achievable, relevant goals (Holmberg Fagerlund et al. 2019; Ishikawa et al. 2023). Which coping responses are acceptable to parents in our study may reflect their cultural norms. For example, care and its expression may differ in how the child's role in feeding is viewed. Western responsive feeding emphasises care as responding to a child's hunger and satiety cues, whereas in many Majority World contexts, care is expressed through the act of providing food (Scheidecker et al. 2025). Therefore, coping responses around supporting child autonomy may not be a ‘both/and’ strategy in cultures from the Majority World. Thus, rather than prescribing one‐size‐fits‐all nutrition advice, professionals and interventions could offer a range of practical coping responses that parents can select according to their values and circumstances. Future research could explore cultural norms in relation to acceptable coping responses, as well as tailoring advice to values and circumstances through professionals (e.g., youth doctors) or targeted interventions. Examples of tailoring suggested in the literature include aligning support with an individual's life goals and priorities (Hoveling et al. 2025), setting realistic goals that account for environmental barriers (Coupe et al. 2018), and providing advice that considers food cost concerns (Bukman et al. 2014). Nonetheless, some existing advice was considered helpful, as participants found resources on behavioural management, recipes, and picky eating useful for coping with their children's nutrition‐related behaviours. We speculate that this advice was helpful because it fit their context and led to desirable outcomes for parents regarding their values.

### 4.3 Addressing Barriers and Support for Healthy Coping

Nutrition literature often frames coping as an individual responsibility, placing pressure on parents to manage challenges alone despite experiencing structural barriers (Jovanovski and Cook 2019). Our findings, however, provide evidence that time, material resources, and social support are crucial for facilitating ‘both/and’ coping strategies. Key facilitators include assistance with caregiving, cooking, and grocery shopping; support in maintaining routines through daycare and school; and practical information, such as recipes and tips for managing food rejection.

The high prevalence of trade‐off coping among our participants in disadvantaged contexts underscores the need to make ‘both/and’ coping feasible in circumstances that include poverty and limited time, influenced by work conditions or social services. Trade‐offs can be a reasonable response to these constraints (Frankenhuis and Nettle 2020). Prior research also confirms that social support reduces strain and helps maintain healthy nutrition (Benson et al. 2024; Ryu 2024). We recommend that nutrition interventions expand coping resources through social support, thereby changing the person–environment relationship that drives and can resolve distress.

Beyond social support, we suggest that policymakers and intervention designers address employment conditions, childcare access, income security, and neighbourhood resources, such as safe play spaces and proximity to affordable, healthy food options. These conditions are all underlying or directly related to what participants identified as supporting ‘both/and’ coping responses for healthy eating for their child. Recognising how these contextual factors shape coping choices allows us to move from judging “right” responses toward understanding what is feasible within parents' circumstances and adequately supporting nutritional choices of families at the neighbourhood, city, and national levels.

### 4.4 Limitations

Several limitations should be noted. The interviews primarily focused on challenging moments and how parents managed them. Although we asked what went well and what parents felt proud of, our interview focus likely resulted in more unresolved situations being discussed than if the study had focused mainly on successfully resolved challenges. Additional 'both/and' responses might have emerged with a narrower focus on helpful coping. Therefore, the responses should be considered exploratory rather than definitive. Another limitation was the use of visual and verbal prompt cards. While intended to encourage thoughtful answers, they may have steered conversations toward situations and challenges in the nutrition literature, limiting spontaneous insights. To mitigate this, interviews used open‐ended questions and prompt cards with general terms and images (e.g., ‘social media’ or a moon depicting evening and nighttime) to explore what was experienced as supportive. These open‐ended cards may have encouraged more discussion of ‘both/and’ coping that reduces tensions, rather than only discussing current challenges and ‘either/or’ coping that leaves tensions intact. In two cases, a translation app and an interpreter were used, which may have affected dialogue depth, nuances in interpretation, and transcription accuracy. Finally, some interviews took place in public settings, and participants' children were often present. This may have limited privacy and affected responses. For example, passersby may have overheard responses, or responses may have been less detailed because participants' attention was split between the interview and their child. However, participants were asked to choose interview locations where they felt comfortable; therefore, any public settings were selected by the participants themselves.

## 5 Conclusion

This study explored how parents of children aged 0–5 in disadvantaged neighbourhoods cope with value tensions in nutrition decisions for their children and how their context influences their coping responses. Interviews revealed that parents employ diverse coping responses to manage value tensions between their child's nutritional health and non‐health values, supporting their own well‐being. While often trade‐off coping strategies were used when one value was prioritised, ‘both/and’ strategies offer promising avenues for addressing distress in parents and supporting healthy eating in children. These strategies not only alleviate distress from neglecting a critical value but can also change the conditions that create the value tension. Three themes of ‘both/and’ coping responses were identified: planning and preparing food, managing stressful child behaviours and supporting children's healthy food acceptance. The feasibility of actualising these responses depended on resources and social support, underscoring the need for interventions and policies that address structural barriers and expand opportunities for healthy ‘both/and’ coping. Coping facilitators operated across socio‐ecological levels, including financial resources at the individual level, caregiving support and help with cooking and shopping at the interpersonal level, and access to safe neighbourhood play spaces at the community level. Overall, these findings indicate that parents can make healthy nutrition choices while navigating stress from value tensions, highlighting how value‐sensitive, context‐aware interventions could further create opportunities for ‘both/and coping’.

## Author Contributions

N.B., M.B.V., and V.T.V. designed the research project. V.T.V. and M.B.V. also supervised the overall study. N.B. was involved in creating materials for the methods, conducted participant recruitment, and executed data collection. N.B. was involved in data analysis, in consultation with V.T.V. and M.B.V. N.B. drafted the manuscript in close collaboration with V.T.V. and M.B.V. All authors read, edited and approved the final version of the manuscript. V.T.V. and M.B. acquired financial support for the project that led to this publication.

## Conflicts of Interest

The authors declare no conflicts of interest.

## Supporting information

Supporting File 1.

Supporting File 2.

## Data Availability

The data that support the findings of this study are available on request from the corresponding author. The data are not publicly available due to privacy or ethical restrictions.
